# Cross species comparison of C/EBPα and PPARγ profiles in mouse and human adipocytes reveals interdependent retention of binding sites

**DOI:** 10.1186/1471-2164-12-152

**Published:** 2011-03-16

**Authors:** Søren F Schmidt, Mette Jørgensen, Yun Chen, Ronni Nielsen, Albin Sandelin, Susanne Mandrup

**Affiliations:** 1 Department of Biochemistry and Molecular Biology, University of Southern Denmark, Campusvej 55, DK-5230 Odense M, Denmark; 2 The Bioinformatics Centre, Department of Biology and Biomedical Research and Innovation Centre, Copenhagen University, Ole Maaløs Vej 5, DK-2200, Copenhagen N, Denmark

## Abstract

**Background:**

The transcription factors peroxisome proliferator activated receptor γ (PPARγ) and CCAAT/enhancer binding protein α (C/EBPα) are key transcriptional regulators of adipocyte differentiation and function. We and others have previously shown that binding sites of these two transcription factors show a high degree of overlap and are associated with the majority of genes upregulated during differentiation of murine 3T3-L1 adipocytes.

**Results:**

Here we have mapped all binding sites of C/EBPα and PPARγ in human SGBS adipocytes and compared these with the genome-wide profiles from mouse adipocytes to systematically investigate what biological features correlate with retention of sites in orthologous regions between mouse and human. Despite a limited interspecies retention of binding sites, several biological features make sites more likely to be retained. First, co-binding of PPARγ and C/EBPα in mouse is the most powerful predictor of retention of the corresponding binding sites in human. Second, vicinity to genes highly upregulated during adipogenesis significantly increases retention. Third, the presence of C/EBPα consensus sites correlate with retention of both factors, indicating that C/EBPα facilitates recruitment of PPARγ. Fourth, retention correlates with overall sequence conservation within the binding regions independent of C/EBPα and PPARγ sequence patterns, indicating that other transcription factors work cooperatively with these two key transcription factors.

**Conclusions:**

This study provides a comprehensive and systematic analysis of what biological features impact on retention of binding sites between human and mouse. Specifically, we show that the binding of C/EBPα and PPARγ in adipocytes have evolved in a highly interdependent manner, indicating a significant cooperativity between these two transcription factors.

## Background

The adipose tissue plays a central role in maintaining whole body lipid and glucose homeostasis as well as insulin sensitivity [1,2]. Adipocytes are derived from fibroblastic precursors in the adipose tissue through a tightly regulated differentiation process. The molecular basis for the regulation of adipogenesis has been studied extensively *in vitro *using a variety of preadipocyte cell culture models. In particular, studies of the 3T3-L1 cell line derived from mouse embryo fibroblasts [3] have been valuable for gaining insight into the ordered cascade of molecular events required for adipogenesis [4-6]. More recently, human cell culture models have become available including the SGBS cell line derived from preadipocytes from a patient with Simpson-Golabi-Behmel syndrome [7].

The transcription factor peroxisome proliferator activated receptor (PPARγ) is a key regulator of adipogenesis, required for *in vitro *as well as *in vivo *differentiation of adipocytes [8,9]. In addition to PPARγ, a number of other important transcriptional regulators of adipocyte differentiation have been identified [6], including members of the CCAAT/enhancer binding protein (C/EBP) family [10-12]. C/EBPα is induced late in adipocyte differentiation and is known to cooperate with PPARγ in induction of at least a subset of adipocyte-specific genes. In addition, these two factors induce the expression of each other [13,14]. Two other members of the C/EBP family, C/EBPβ and -δ, are directly involved in the transcriptional induction of PPARγ and C/EBPα [6].

We recently used chromatin immunoprecipitation (ChIP) combined with deep sequencing (ChIP-seq) to generate genome-wide maps of the binding sites of PPARγ and its heterodimerization partner retinoid × receptor (RXR) during differentiation of 3T3-L1 adipocytes [15]. In addition, we profiled RNA polymerase II (RNAPII) occupancy to measure active transcription at different time points during differentiation. This study revealed that PPARγ:RXR binding was highly enriched in the vicinity of genes upregulated during adipogenesis. In fact, the majority (75%) of all highly up-regulated genes have PPARγ:RXR binding in the immediate vicinity [15]. Similarly, Lazar and colleagues [16] and others [17,18] used ChIP in combination with hybridization to genomic tiling microarrays (ChIP-chip) or cloning followed by sequencing (ChIP-PET) to map PPARγ binding sites in 3T3-L1 adipocytes (reviewed in [19]). Intriguingly, these studies have revealed that the C/EBPα consensus site is highly over-represented under the binding regions of PPARγ. Lazar and colleagues profiled C/EBPα binding sites in mature 3T3-L1 adipocytes and found a remarkable overlap between C/EBPα and PPARγ binding (> 60% of all PPARγ sites) on a genome-wide scale [16]. Importantly, 60% of the genes induced during adipogenesis have both C/EBPα and PPARγ binding sites within 50 kb of a transcription start site (TSS), and knockdown studies indicated that both C/EBPα and PPARγ are required for robust gene expression of a few selected adipocyte specific target genes. Cumulatively, these results indicate that both PPARγ and C/EBPα are directly involved in the activation of the majority of adipocyte-specific genes and that they cooperate through binding to adjacent sites on DNA.

Genome-wide profiling has also made it possible to study the evolution of gene regulation by mapping the sites for the same transcription factors in different species. This is typically done by aligning genomes of the two species and tabulating the number of detected sites in one species that are in the corresponding region in the other species. This means that a genomic region might be highly conserved in terms of nucleotides but may or may not bind the transcription factor in question in both species. To distinguish between genome sequence conservation and transcription factor binding site conservation, we will use the word "retention" to describe binding sites that are present in both species in the corresponding genomic region.

Notably, whereas the functional gene targets of a particular transcription factor are generally well conserved between species, it has been shown that the majority of binding sites for all transcription factors investigated to date are species-specific [20-25], reviewed in [26]. This is surprising given that sequence conservation has been successfully used to enhance regulatory site prediction in proximal promoters (phylogenetic foot printing) [27,28]; however, this might reflect that these older studies were focused on limited sets of sites often located in tissue-specific promoters, while genome-wide methods, such as ChIP-seq, are independent of previous annotations. Consistent with the species specificity of transcription factor binding sites, Rosen and colleagues recently compared PPARγ binding in 3T3-L1 adipocytes and *in vitro *differentiated primary human adipose stromal cells (hASC), and found that only 21.3% of the murine binding sites were retained in human adipocytes. By contrast, the association of PPARγ with adipocyte gene regulation appeared to be better retained than the specific binding sites, and the overall gene expression profiles were well conserved [21]. Rosen and colleagues also showed that genes associated with a conserved PPARγ binding site are more likely to be upregulated during adipogenesis than genes associated with a species specific site [21], indicating that retention of PPARγ binding is increased near upregulated genes.

While previous reports agree that retention of transcription factor binding sites is limited, systematic analysis of the biological features determining whether a site is retained or not has not been performed. In particular, the interdependence between retention of the transcription factor binding sites of two transcription factors has not been investigated. Here, we used ChIP-seq to generate genome-wide binding profiles of C/EBPα and PPARγ in human SGBS adipocytes, compared these to previously published profiles in mouse 3T3-L1 adipocytes [15,16], and systematically studied what features affect whether a binding site is retained between species or not.

We find that PPARγ binding sites have higher retention near genes upregulated during adipogenesis, and that regions bound by both factors are even more likely to be retained. Interestingly, PPARγ binding site retention in these co-bound regions is increased by the presence of a C/EBPα consensus site, suggesting that C/EBPα may facilitate PPARγ binding to DNA. At the same time, and independent of C/EBPα and PPARγ sequence patterns, sequence conservation in the larger region surrounding the actual binding sites has a positive impact on retention of both C/EBPα and PPARγ binding sites, indicating that other DNA sequence patterns also affect binding of these two factors to DNA.

## Results

### Genome-wide mapping of C/EBPα and PPARγ binding in human SGBS adipocytes

To compare C/EBPα and PPARγ binding in mouse and human adipocytes, we used ChIP-seq to generate genome-wide profiles of C/EBPα and PPARγ binding in human SGBS adipocytes. Mapped ChIP tags were analyzed using peak finder methods, which identify regions enriched for ChIP tags. For simplicity, we will refer to such regions as "sites", but it is important to notice that these regions are typically 100-400 nt wide (up to 1000 nt wide in ChIP-chip studies) and thus several magnitudes larger than the actual binding site (typically 5-20 nt). We used the MACS peak finder [29] to identify regions consistently bound by C/EBPα and PPARγ in two biologically independent experiments. We detected 52,733 C/EBPα and 23,328 PPARγ binding sites in human SGBS adipocytes, and in accordance with previous findings in mouse [16], we found that 49.5% of PPARγ binding sites are also bound by C/EBPα. The number of PPARγ sites is comparable to the 39,968 sites recently reported by Rosen and colleagues for in vitro differentiated hASCs [21]. However, the numbers are notably higher than the corresponding numbers in 3T3-L1 cells, where 16,760 [16] and 15,461 (this study) C/EBPα sites and 5299 [16] and 6952 [15] PPARγ sites were reported. It remains to be investigated whether this represents a true species-specific difference, or whether this is more related to technical issues such as antibody specificity or cell culture conditions.

**Figure 1:**
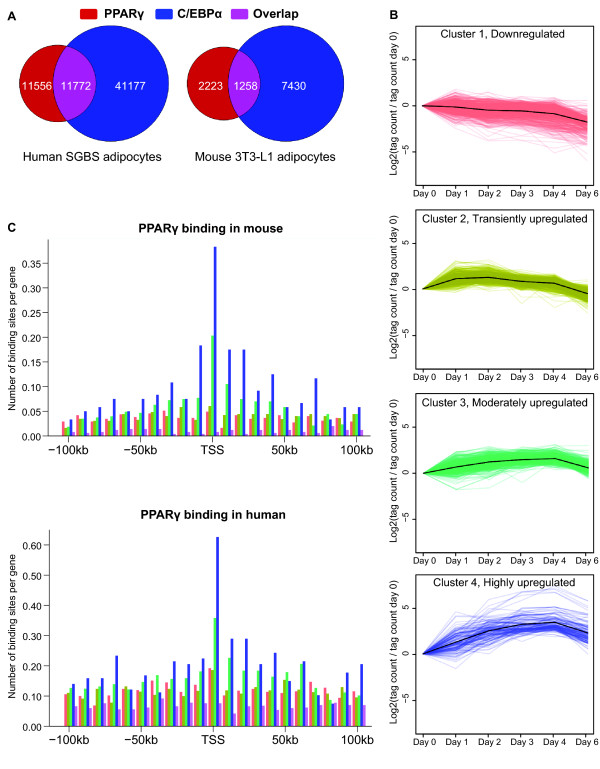
**Genome-wide mapping of C/EBPα and PPARγ binding in human SGBS adipocytes**. **(A) **The number of C/EBPα and PPARγ sites in SGBS and 3T3-L1 adipocytes. Results are shown as Venn diagrams representing the number of binding sites for C/EBPα only (blue), PPARγ only (red), and overlapping binding sites (purple). **(B) **Expression clusters of genes based on their changes in RNAPII occupancy during 3T3-L1 adipogenesis. The clusters correspond to genes that are (1) downregulated, (2) transiently upregulated, (3) moderately upregulated or (4) highly upregulated. **(C) **Distribution of PPARγ sites relative to TSS of downregulated genes (magenta), transiently upregulated genes (yellow), moderately upregulated genes (green), highly upregulated genes (blue) and randomly selected regions (purple), in mouse and human. The region +/- 100 kb around each TSS was divided into 10 kb regions, and the mean number of sites in each region is indicated in the bar plots.

We have previously reported that binding profiles of PPARγ in 3T3-L1 s vary significantly between individual experiments and laboratories [19]. Similarly, when comparing the PPARγ sites of our own ChIP-seq study [15], the ChIP-seq study of Rosen and colleagues [21], and the ChIP-Chip study by Lazar and colleagues [16] only approximately 50% of the detected peaks were shared between 2 experiments, and only 2025 of a total of 12,136 peaks (16,6%) were shared between all 3 experiments (Additional file 1 Fig. S1). To infer high confidence binding sites occupied by C/EBPα and PPARγ in mouse adipocytes, we used only the 8688 C/EBPα and 3481 PPARγ binding sites from our ChIP-seq analysis that overlap with the previously published ChIP-chip sites [16]. The inferred high confidence C/EBPα and PPARγ binding sites have a smaller overlap of 36% (Figure 1A), compared with the previously reported >60% [16]. Possible explanations may be the higher resolution of the ChIP-seq data as well as the fact that the number of high confidence sites used is lower.

### Correlating binding sites with gene expression patterns during adipogenesis

We previously identified 1650 genes which were differentially expressed during 3T3-L1 adipogenesis as assessed by RNAPII association to the respective genes [15]. In that study, these genes were clustered in 5 different expression clusters depending on their relative expression profile during differentiation. For the purpose of this study we combined the two clusters containing genes downregulated during 3T3-L1 adipogenesis. This resulted in four new clusters: downregulated (1), transiently upregulated (2), moderately upregulated (3), and highly upregulated (4) (Figure 1B). For each gene belonging to a particular cluster, we divided the genomic region at +/- 100 kb around TSS into 10 kb intervals ("bins") and counted the number of mouse PPARγ binding sites within each such intervals. The PPARγ binding sites are enriched in the vicinity of all regulated genes compared to randomly selected regions (Figure 1C). Moreover and consistent with previous findings [15,16], PPARγ binding sites in mouse adipocytes are highly enriched in the vicinity of genes moderately (3) and highly (4) upregulated during adipogenesis (Figure 1C). Similarly, PPARγ binding sites are also enriched in the vicinity of the human gene orthologs of the genes in these clusters (Figure 1C), consistent with a role for PPARγ in the regulation of these genes in SGBS adipocytes.

### Measuring retention of mouse binding sites in human

To study the retention of C/EBPα and PPARγ binding sites between mouse and human adipocytes, we used human-mouse genome alignments [30] to identify human genome regions orthologous to C/EBPα and PPARγ sites in mouse (see Methods). To make sure that the sites were orthologous at genome sequence level, a mouse site had to map to a single location in the human genome, which in turn had to map back to the original site in mouse. We did not address site loss or gain due to large deletions or insertions in one species. This resulted in 2176 PPARγ and 4899 C/EBPα mouse sites that could be mapped to the human genome (62.5% and 56.3% of all detected mouse PPARγ and C/EBPα binding sites, respectively).

**Figure 2:**
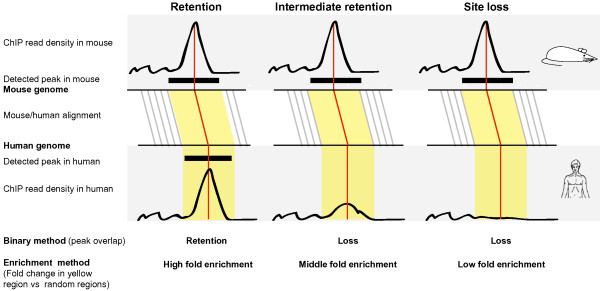
**Two methods for measuring retention of binding sites**. We use three examples to illustrate our two complementary methods and their detection of retention or loss of mouse sites. Both methods start out from mouse binding sites, detected as peaks from the mouse ChIP-seq data [15]. Red lines show the midpoints of mouse sites and the corresponding positions in the human genome. Similarly, the yellow area corresponds to the ends of the mouse site and the corresponding orthologous sequences in human. **(A) **Retention of a mouse site. The mouse binding region overlaps with a human binding region (peak detected from human data) in the corresponding human orthologous region. Using the binary method, this defines a retained site. The enrichment method compares the ChIP density in the orthologous human region with random regions to calculate fold enrichment. In this case the human ChIP signal is strong which results in a high fold enrichment. **(B) **Intermediary retention. In this example, a fraction of the binding activity remains in the human orthologous region. This will not be detected using peak finding, and the site will be classified as lost using the binary method. However, the enrichment method will give this region an intermediary fold enrichment score, since there are more ChIP-seq tags than expected by random. **(C) **Loss of a mouse site. Here, there is no detectable ChIP-seq signal in the orthologous human region. The binary method will classify this site as non-retained and thereby lost in human, while the enrichment will report a fold enrichment near zero, since the enrichment is similar to that of random regions.

We defined a mouse binding site as retained when its corresponding human coordinates overlapped a human binding site (Figure 2). A potential problem with this approach is that it will be dependent on the thresholds used in peak finding, and it also models average transcription factor binding in a cell population as on-off events, which is a large simplification. Since the relative number of ChIP'ed sequence tags in a region will indicate the average "strength" of the binding, i.e. average occupancy time, we also measured retention in a complementary way. Thus, in a human genomic region corresponding to a mouse binding site, we counted the number of ChIP'ed tags from the transcription factor in question and calculated a log(fold-change) enrichment relative to the genomic background in human (see Methods)(Figure 2). The advantage of this approach compared to the above method is that the retention of sites will not be on-off (binary) events but a continuous measure, and weaker cases of retention may be discovered. We will refer to these two methods to measure the amount of site retention as the binary and enrichment method, respectively.

**Figure 3:**
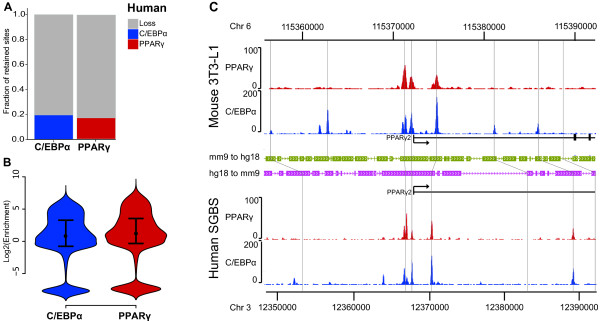
**Limited retention of mouse PPARγ and C/EBPα binding sites**. **(A) **Bar diagrams representing the fraction of retained C/EBPα sites (blue), retained PPARγ binding sites (red), and lost binding sites (gray), all from mouse to human using the binary method. **(B) **Retention using the enrichment method. The plot shows the distribution of tag enrichment in the human regions orthologous to the mouse sites, calculated as log_2_(fold change compared to random expectation). High values indicate high levels of enrichment and therefore retention. C/EBPα and PPARγ retention is indicated by blue and red color, respectively. The distributions are shown as a variant of boxplots called "violin plots", a rotated density plot. The width of the colored area corresponds to how many data points that fall into that range [48]. The black dot within each plot represents the median and hinges the 75^th^, and 25^th ^quartiles of the respective distribution, similar to a box plot. In both cases most of the probability density is located around a log fold change of 0, indicating that most regions are not retained. **(C) **Example of retention of multiple mouse/human sites in the *PPARG2 *locus. The ChIP-seq density is shown for PPARγ (red) and C/EBPα (blue) in both species, together with the UCSC gene models. The *PPARG2 *locus with TSS and exons is indicated as a black line and filled bars, respectively. Grey lines show center in mouse sites and corresponding regions in human. Green and pink arrow structures represent alignment blocks from the UCSC browser.

Using the binary method, we find that most binding sites are species specific; only 19.5% of C/EBPα and 16.9% of PPARγ binding sites in mouse adipocytes are retained in human adipocytes (Figure 3A). The enrichment analysis supports the binary analysis in that most of the enrichment values for both factors are located around 0, which is expected by random, and in that many of the mouse sites map to human regions that are totally devoid of ChIP-seq tags (negative fold change) (Figure 3B).

Conversely, there are sites that we with high confidence can label as retained between species. We noticed that there are larger genomic regions in which the majority of C/EBPα and PPARγ binding are retained as exemplified by the *PPARG2 *locus (Figure 3C). Given the crucial role of PPARγ in adipocyte differentiation, it is tempting to suggest that the reason that these particular sites are retained is their importance for the overall regulatory regime, since they likely regulate a key transcription factor. Prompted by this observation, we proceeded to systematically identify biological features that are positively correlated with increased retention of PPARγ and C/EBPα sites, with the hope of increasing our understanding of adipocyte gene regulation.

### Retention of PPARγ binding sites is increased in the vicinity of genes up-regulated during 3T3-L1 adipogenesis

Several studies have reported that retention of binding sites is generally higher in the vicinity of putative key target genes [20-25]. However, species-specific binding sites are also enriched in these regions, and no studies have addressed whether the relative retention of binding sites (i.e the fraction of the sites in a mouse region that is retained vs. lost in human) is increased in the vicinity of putative target genes of the transcription factor. The assignment of target genes for binding sites is not trivial. Most studies agree that sites proximal to a gene are more likely to regulate the gene in question but this is not an absolute truth. In light of this, we used a combination of distance and gene expression to associate genes and binding sites. Thus, to systematically investigate how binding site retention is affected by the vicinity to putative target genes, we labeled sites in the following way. First, for each binding site, we located the closest gene TSS. If this TSS was closer than 100 kb, and the gene was within one of the pre-defined expression clusters shown in Figure 1 (a differentially expressed gene), the site was labeled with the corresponding cluster number (cluster 1 to 4). Sites where the closest TSS was closer than 100 kb but the gene was not part of any expression cluster (a gene with no measurable difference in expression during adipogenesis) were defined to belong to a new cluster, cluster 5 ("constitutive genes"). Finally, all sites with no gene TSS within 100 kb were defined to belong to a new cluster: cluster 6 ("distal sites"). There are obviously many ways to assign binding sites to putative target genes. However, the overall results described below are not specific to the cluster assignment method used. For instance, assigning sites to expression clusters only based on the identity of the closest gene gave very similar results (data not shown).

**Figure 4:**
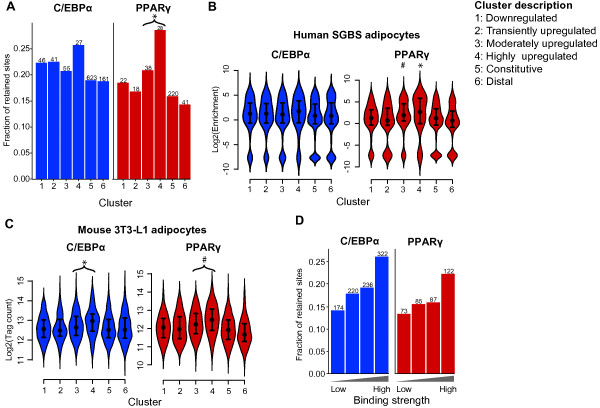
**Binding site retention is correlated with binding strength and vicinity to adipogenic genes**. C/EBPα and PPARγ binding sites were assigned to the closest TSS of a cluster gene within 100 kb. Cluster 5 contains binding sites assigned to genes with no significant expression change, and cluster 6 contains binding sites >100 kb from the closest gene. **(A) **Bar diagrams representing the fraction of retained C/EBPα (blue) and PPARγ (red) bars, broken up by assigned expression cluster. The numbers above the bars represent the total number of retained binding sites in that cluster. *P = 2.2*10^-16 (clusters 3+4 vs. all other clusters (Fisher Exact Test)). **(B) **Complementary violin plots (see Fig. 3B for explanation) showing the distribution of tag enrichment in orthologous human regions, broken up by expression cluster, C/EBPα in red and PPARγ blue. *P = 2.9*10^-5 (cluster 4 vs. all clusters (Wilcoxon test)); #*P *= 4.55*10^-5 (cluster 3 vs. clusters 1+2+5+6 (Wilcoxon test). **(C) **Violin plots showing the distribution of tag counts in mouse binding regions, broken up by expression cluster, C/EBPα in red and PPARγ blue. *P = 1.643*10^-14 (clusters 3+4 vs. all other clusters (Wilcoxon test)); #*P=*5.315*10^-6 (clusters 3+4 vs. clusters all other clusters (Wilcoxon test)) (**D**) Correlation between retention of mouse sites in human and binding strength in mouse measured by the number of ChIP-seq tags. Mouse binding sites were divided into four groups from low to high ChIP-seq density (x-axis), and we investigated what fraction of these sites were retained in human (y axis). Numbers above the bars correspond to the total number of retained mouse sites in human.

We then measured the fraction of mouse C/EBPα and PPARγ binding sites that was retained and lost in human. The fraction of retained PPARγ binding sites is significantly higher in the vicinity of genes that are moderately or highly upregulated (clusters 3 and 4) compared to all other binding sites (Fischer Exact Test, *P *< 2.2*10^-16). Conversely, retention of C/EBPα sites appears to be slightly increased in the vicinity of highly upregulated genes (cluster 4) compared to all other binding sites, but the difference is not statistically significant (Fischer Exact Test, *P *= 0.1) (Figure 4A). This is also true if assessing retention by the enrichment method (Wilcoxon test; C/EBPα, cluster 4 vs. all other clusters, *P *= 0.056; PPARγ cluster 4 vs. all other clusters, *P *= 2.9*10^-5; PPARγ cluster 3 vs. clusters 1+2+5+6, *P *= 4.55*10^-5) (Figure 4B). Further analysis of the enrichment values showed that also mouse C/EBPα and PPARγ binding sites associated with moderately and highly upregulated genes (cluster 3 and 4) have on average more ChIP tags (i.e. display stronger binding) than other sites (Wilcoxon test; C/EBPα, *P *= 1.643*10^-14; PPARγ, *P=*5.315*10^-6) (Figure 4C). Thus, target sites in the vicinity of cluster 4 genes are on average bound more strongly by C/EBPα and PPARγ than sites in the vicinity of other genes (Figure 4B and 4C). This raised the question whether the binding strength in mouse, as measured by the number of ChIP tags, is indicative of binding site retention in human. Therefore, we divided mouse binding sites into quartiles according to binding strength and analyzed retention of C/EBPα and PPARγ binding in these subgroups (Figure 4D). Indeed, mouse sites that are strongly bound by either factor are more likely to be retained in human.

### Turnover of PPARγ binding is increased in the vicinity of genes upregulated during 3T3-L1 adipogenesis

**Figure 5:**
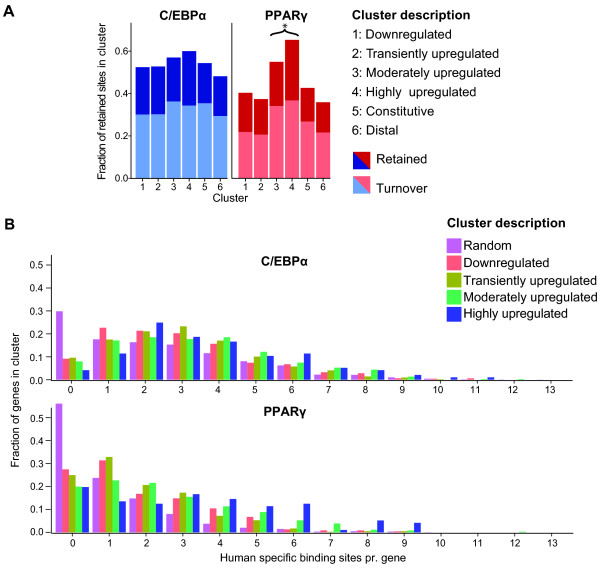
**Turnover of binding sites is increased in the vicinity of adipogenic genes**. **(A) **Bar diagrams representing the fraction of transcription factor binding turnover events (C/EBPα: light blue, PPARγ: light red) and retained binding sites (C/EBPα: blue, PPARγ: red) broken up by expression clusters as in Fig. 4. *P=8.183*10^-4 (clusters 3+4 vs. all other clusters (Wilcoxon test) **(B) **Histograms illustrating the fraction (y axis) of human genes within a particular cluster that have the indicated number (x axis) of human-specific binding sites within 50 kb of the TSS.

As discussed above, most mouse sites are not retained in human. However, gene regulation may be retained, if a lost site is replaced by another species-specific site in human nearby the "original" mouse site [31]. We will refer to this scenario as site turnover. Here we defined turnover as a case where a mouse binding site is lost at the corresponding region in human, but compensated by a human-specific site within 10 kb of the location of the lost site in human. Employing these criteria, we analyzed turnover of C/EBPα and PPARγ binding sites assigned to the different gene expression clusters. Interestingly, the turnover of PPARγ binding sites is highly increased for sites close to moderately or highly upregulated genes (clusters 3 and 4) (Wilcoxon test, *P=*8.183*10^-4) (Figure 5A). In particular, >60% of PPARγ binding sites in the vicinity of highly upregulated genes in mouse adipocytes are either retained or undergo turnover in human adipocytes. Moreover, the highly upregulated genes are much more likely than genes in other clusters to gain multiple human specific PPARγ binding sites in human adipocytes (Figure 5B). By contrast, human-specific C/EBPα sites are found more evenly distributed in the different clusters (Figure 5A+B).

### Retention of C/EBPα and PPARγ binding is increased at regions bound by both C/EBPα and PPARγ

**Figure 6:**
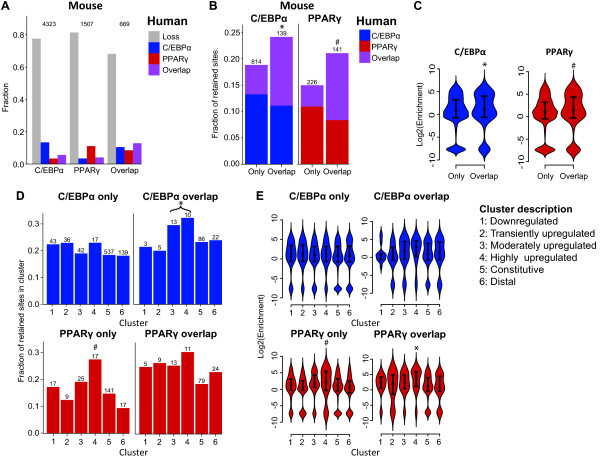
**PPARγ/C/EBPα overlap increases retention of binding sites**. **(A) **Bar diagrams illustrating the fraction of C/EBPα only, PPARγ only and overlapping PPARγ-C/EBPα binding sites (x axis) in mouse mouse that are either lost (gray), or found as a C/EBPα only (blue), PPARγ only (red) or PPARγ-C/EBPα (purple) binding site in human adipocytes. Numbers above the bars correspond to the total number of mouse sites in each group. **(B) **Retention of mouse C/EBPα and PPARγ binding sites in mouse adipocytes (x axis) in the corresponding human regions. The bars represent fractions of mouse binding sites retained as C/EBPα-only (blue), PPARγ-only (red), and overlap (purple) binding sites in human. The x axis breaks up each set of transcription factor binding sites according to, whether it is C/EBPα- or PPARγ-only in mouse, or whether the site is also overlapped with the other factor in mouse.*P = 3.5*10^-3 (C/EBPα overlap vs. C/EBPα only (Fisher Exact Test)); #*P *= 6.2*10^-4 (PPARγ overlap vs. PPARγ only (Fisher Exact Test)). **(C) **Violin plots (see Fig. 3B for explanation) showing the distribution of tag enrichment in orthologous human regions, broken up depending on whether a site is overlapped or not in mouse adipocytes as in Fig. 6B. *P = 0.007 (C/EBPα overlap vs. C/EBPα only (Wilcoxon test)); #*P *= 3.9*10^-4 (PPARγ overlap vs. PPARγ only (Wilcoxon test)) **(D) **Retention of C/EBPα-only or PPARγ-only and PPARγ-C/EBPα binding sites in mouse, broken up by expression clusters. *P = 8.6*10^-15 (clusters 3+4 vs. all other clusters (Fisher Exact Test)); #P = 0.01 (cluster 4 vs. all other clusters (Fisher Exact Test)). **(E) **Violin plots showing the distribution of tag enrichment in orthologous human regions, broken up depending on whether a site is overlapped or not and on associated gene cluster in mouse adipocytes. #*P *= 8.072*10^-3 (cluster 4 vs. All clusters (Wilcoxon test)); *P = 0.001 (cluster 4 vs. all other clusters (Wilcoxon test)).

As discussed above, a large fraction of PPARγ binding sites (36%) are also bound by C/EBPα. To assess the importance of this overlap, we compared the retention of mouse sites that consist of either: i) overlapping PPARγ and C/EBPα sites, ii) a single PPARγ site or iii) a single C/EBPα site (Figure 6A-B). We will refer to these cases as 'overlapping sites' and PPARγ- or C/EBPα-only sites, respectively. The sites in the overlapping group are retained to a higher degree than the other two categories using the binary method (Fisher Exact Test; PPARγ, *P *= 6.2*10^-4; C/EBPα, *P *= 3.5*10^-3) (Figure 6B); and this is also true if the enrichment method is used (Wilcoxon test; PPARγ, *P *= 3.9*10^-4; C/EBPα, *P *= 0.007) (Figure 6C). Interestingly, the overlapping sites are primarily retained as overlapping sites in human, whereas PPARγ-only and C/EBPα-only sites are primarily retained as such (Figure 6A-B). However, a few percent of PPARγ and C/EBPα-only sites in mouse are detected as overlapping sites in human. We cannot exclude that some of these could be the result of threshold issues in the mouse dataset.

Since we now established that both the expression pattern of nearby genes and co-binding of PPARγ and C/EBPα have an impact on retention of sites, we proceeded to see how these two associations are depending on one another. About 20% of C/EBPα-only sites are retained in human, regardless of expression pattern of nearby genes (Figure 6D), but C/EBPα binding sites overlapping PPARγ sites have a significantly higher retention if they are in the vicinity of moderately or highly upregulated genes (cluster 3 and 4) (Fisher Exact Test, *P *= 8.6*10^-15). Thus, the increased retention of C/EBPα binding in the vicinity of highly upregulated genes (Figure 4A), originates from increased retention of the binding sites that overlap with PPARγ binding.

For PPARγ-only sites there are large differences in the amount of retention depending on which gene expression cluster the sites are assigned to, with sites close to highly upregulated genes most likely to be retained (Fisher Exact Test, *P *= 0.01). By contrast, retention of PPARγ sites overlapping with C/EBPα is less affected by proximity to highly upregulated genes (Fisher Exact Test, *P *= 0.2, PPARγ overlap cluster 4 vs. all other cluster) (Figure 6C). Interestingly, retention of PPARγ sites near highly upregulated genes is not affected by overlap with C/EBPα (Fisher Exact Test, *P *= 0.8179, PPARγ overlap cluster 4 vs. PPARγ only cluster 4). Overall these findings are supported by the enrichment analysis (Figure 6E), although in this case, the enrichment of C/EBPα sites that overlap with PPARγ sites near moderately and highly upregulated genes is only close to significant (Wilcoxon test, *P *= 0.05707, clusters 3+4 vs. all other clusters), and the enrichment of PPARγ sites that overlap with C/EBPα sites near highly upregulated genes is now significantly higher than for other PPARγ sites overlapping C/EBPα (Wilcoxon test, *P *= 0.001).

### C/EBPα sequence patterns are indicative of PPARγ retention in human but not vice versa

**Figure 7:**
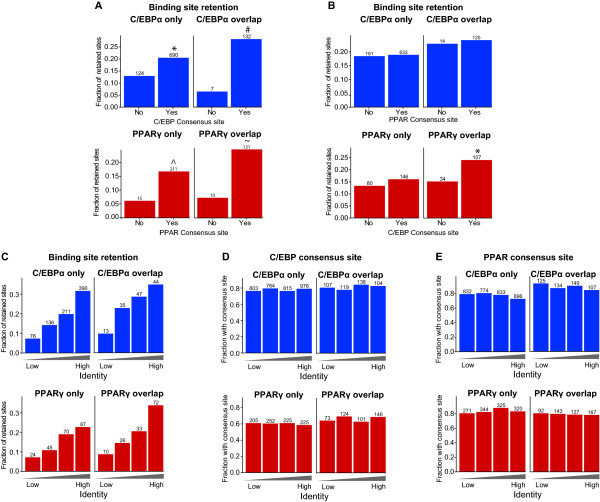
**Correlation between retention, consensus motifs and mouse-human sequence identity**. **(A) **Retention of mouse C/EBPα and PPARγ binding sites in human adipocytes with or without a corresponding consensus site within the orthologous human regions. *P = 8.125*10^-08 (Fisher Exact Test); #*P *= 3.344*10^-07 (Fisher Exact Test); ^*P *= 5.533*10^-06 (Fisher Exact Test); ~*P *= 1.935*10^-06 (Fisher Exact Test). **(B) **Retention of mouse C/EBPα binding sites in human adipocytes with or without a PPARγ consensus site; and conversely, retention of PPARγ binding sites in human adipocytes with or without a C/EBPα consensus site. *P = 0.009 (C/EBPα consensus site vs. no consensus site (Fisher Exact Test)). **(C) **Correlation between binding site retention and overall mouse-human DNA sequence identity of the aligned region. Sites were divided into four groups from lowest to highest mouse-human sequence identity, and the fraction of retained sites in each group is shown on the y axis. **(D) **Fraction of sites having predicted C/EBP consensus sites, broken up by overall sequence identify and whether the sites are overlapped or not. **(E) **Fraction of sites having predicted PPAR consensus sites, broken up by overall sequence identify and whether the sites are overlapped or not. Numbers above the bars correspond to the total number of mouse sites in each group.

To further investigate the interdependence of retention of C/EBPα and PPARγ binding at overlapping sites, we wanted to assess whether the presence of predicted binding sites (consensus sites) for the respective factors in human were indicative of retention of mouse binding sites in human. We scanned each of the human regions orthologous to mouse PPARγ and C/EBPα binding sites with position weight matrices (PWM) (reviewed in [32,33]) describing the DNA binding preference of C/EBPα and PPARγ. We used thresholds defined by simulation studies (see Methods), and noted whether each binding site had one or more consensus sites for the respective factor. For this analysis, it is important to consider that the consensus site for C/EBPα is similar to that of most other members of the C/EBP family. As expected, PPARγ-binding sites in mouse are more likely to be retained in human if the corresponding human region has a PPARγ consensus site, and mouse C/EBPα binding sites are also more likely to be retained if their corresponding human regions have a C/EBP consensus sites (Fisher Exact Test; C/EBPα only, *P *= 8.125*10^-08; C/EBPα overlap, *P *= 3.344*10^-07; PPARγ only, *P *= 5.533*10^-06; PPARγ overlap, *P *= 1.935*10^-06) (Figure 7A).

Interestingly, we found that PPARγ binding sites overlapping C/EBPα sites in mouse are more likely to be retained as PPARγ binding sites in human if they have a C/EBP consensus site (Fisher Exact Test, *P *= 0.009)(Figure 7B). We noticed the same tendency for PPARγ binding sites not overlapping C/EBPα sites in mouse, although this trend is not strong enough to be statistically significant (Fisher Exact Test, P = 0.16). However, the opposite is not true, i.e. PPARγ consensus sites have no impact on the retention of mouse C/EBPα binding sites in human (Fisher Exact, *P *= 0.88) (Figure 6B).

One way to interpret this is that for regions bound by both factors, the C/EBP consensus site is being selected for over evolution rather than the PPARγ consensus site, which would indicate that PPARγ binding is dependent on sequence-specific C/EBP binding. There are several mechanisms that could explain this, including PPARγ binding to suboptimal sites with the help of C/EBPα, indirect binding by tethering to C/EBPα, and indirect binding via long range chromosomal loops [34,35].

It is likely that in addition to C/EBPα, several other transcription factors cooperate with PPARγ in the transactivation of nearby genes, and some of these factors may also cooperate with PPARγ in DNA binding. Such cooperativity would be expected to contribute to the retention of PPARγ sites. We investigated how the evolutionary constraint on the overall DNA sequence within the binding region of each mouse PPARγ and C/EBPα binding site affects retention of these binding sites in human. We found that regions with high mouse-human DNA sequence identity have higher retention of both C/EBPα and PPARγ binding (Figure 7C). Interestingly, the "high identity quartile" is not enriched for regions containing C/EBPα and PPARγ consensus sites (Figure 7D and 7E), indicating that other sequence patterns/transcription factors are also important for retention of C/EBPα and PPARγ binding.

## Discussion

In this study, we used ChIP-seq to generate genome-wide profiles of the binding sites of the major adipogenic transcription factors, C/EBPα and PPARγ, in mature human SGBS adipocytes. We identified 52,733 C/EBPα and 23,328 PPARγ binding sites, and consistent with previous studies in mouse adipocytes [15,16] we found that PPARγ binding sites are highly enriched in the vicinity of genes upregulated during adipogenesis. Furthermore, and also consistent with what has been reported for mouse adipocytes [16], a high percentage (49.5%) of the PPARγ binding sites overlap with C/EBPα binding sites. Thus, these data indicate conservation of the overall regulatory regime of C/EBPα and PPARγ between mouse and human adipocytes, including their potential direct cooperativity through binding to adjacent sites. However, despite the conservation of the overall regulatory regime and putative target genes between mouse and human adipocytes the retention of mouse binding sites in human is limited, i.e. most sites are species-specific. Similar results demonstrating limited retention of binding sites despite extensive conservation of association with putative functional targets have previously been reported for other transcription factors [20,22-25], and was recently also reported by Rosen and colleagues when comparing PPARγ binding sites in primary human in vitro differentiated adipocytes with the binding sites identified in murine 3T3-L1 cells [21].

Despite the fact that most binding sites of PPARγ and C/EBPα are species-specific, there are some sites that we can confidently classify as retained between human and mouse. There are also larger genomic regions, like the *PPARG2 *locus, where most sites are retained (Figure 3C). This observation prompted us to systematically investigate what biological features determine whether a site is retained or not. Thus, we have systematically investigated several biological features that could affect the retention of mouse C/EBPα and PPARγ binding sites, using two complementary methods with different advantages, i.e. a binary approach where overlaps of sites are counted, and an enrichment approach where the signal strength of human ChIP reads is assessed. The binary approach has the advantage of being conservative (i.e. only strong signals are being labeled as bound sites), and it is easier to interpret the outcomes as sites can be either lost or retained. Conversely, the enrichment method can take weaker sites into account and is not dependent on specific thresholds in peak finding; however, this method will not clearly define what sites are retained and lost, as it imposes no thresholds on the data.

Regardless of method, we find that there is a significantly increased retention as well as turn-over of PPARγ binding sites in the vicinity of genes upregulated during adipogenesis compared with binding sites more distant from such genes. By contrast, retention of C/EBPα binding is only slightly increased in the vicinity of genes that are highly upregulated. The difference indicates that there is a higher evolutionary pressure to maintain specific PPARγ binding sites compared with specific C/EBPα binding sites for the regulation of adipocyte differentiation and function. This may reflect the higher importance of PPARγ over C/EBPα in adipocyte differentiation, as most clearly demonstrated by Spiegelmann and colleagues, who showed that that ectopic expression of PPARγ in C/EBPα -/- mouse embryo fibroblasts (MEF) [36], but not C/EBPα in PPARγ -/- MEFs [14], can induce adipogenesis. In addition, since there are more C/EBPα binding sites than PPARγ binding sites there may be less evolutionary constraint on the individual C/EBPα sites. This could reflect the lower binding specificity of C/EBPα, since it is easier to create a new site if the factor can bind to many different sequences.

We also observed that mouse sites that are strongly bound by either factor are more likely to be retained, but the causality is unclear. Stronger binding sites could be under higher selective pressure, since they are more likely to have a vital function. In addition, stronger binding sites could be harder to erode over time, as these are likely to represent sites where multiple factors such as co-binding transcription factors and chromatin accessibility contribute to a high occupancy of the site. Thus, strongly bound sites in a common ancestor between two species might require more evolutionary changes to be reduced to a fully inactive site in one species.

The remarkable overlap between PPARγ and C/EBPα binding reported previously [15,16] indicates cooperativity between the two transcription factors. In support of this, we find that retention of both C/EBPα and PPARγ binding is increased, when the binding sites overlap with a binding site of the other factor, suggesting that a potential synergy between these two factors is of such significance that selective pressure on these overlapping sites is increased. Interestingly, while retention of C/EBPα-only sites is not affected by vicinity to regulated genes, the C/EBPα sites that overlap with PPARγ sites display increased retention in the vicinity of upregulated genes, further indicating an importance of C/EBPα co-binding with PPARγ in adipocyte gene regulation.

We also analyzed the sequence of human genomic regions corresponding to overlapping PPARγ and C/EBPα binding sites in mouse adipocytes. These analyses revealed that presence of the C/EBP consensus sequence in the human sequence not only predicts the retention of C/EBPα binding but, surprisingly, also retention of PPARγ binding at sites that overlap with C/EBPα binding sites in mouse. By contrast, the PPAR consensus sequence in the human sequence only predicts retention of PPARγ binding, not retention of C/EBPα binding at sites overlapping with PPARγ binding sites. This indicates that at these overlapping sites, the C/EBP consensus sequence in the binding region is being selected for over evolution to a higher degree than the PPAR consensus site, suggesting that PPARγ binding is directed by C/EBPα binding. By analogy, differential binding of nuclear factor κB (NFκB) in 10 human individuals correlates with changes in a consensus sites of a reported cooperating transcription factor, signal transducer and activator of transcription 1 (Stat 1) [37], and similar results have been found in studies in yeast [38] and fungi [39].

Possible mechanisms explaining the interdependence between observed C/EBP consensus site conservation and PPARγ retention include C/EBP-assisted binding of PPARγ to an adjacent consensus site as well as indirect binding of PPARγ to DNA by tethering to C/EBPα or by long range intrachromosomal loops [34,35]. Lazar and colleagues have previously investigated the interdependence of C/EBPα and PPARγ binding in mature 3T3-L1 adipocytes and failed to see an effect of C/EBPα knockdown on PPARγ binding to selected target sites in mature 3T3-L1 adipocytes [16]. However, since only a few sites were investigated it is difficult to conclude on the general importance. Furthermore, it is possible that other members of the C/EBP family, which all share the same consensus sequence, play a role in the establishment of the PPARγ transcriptional complex during adipogenesis and a more limited role once the complex is established. Intriguingly, recent data from our laboratory indicate that C/EBPβ, which is expressed early in differentiation, may play a role in early chromatin remodeling of PPARγ binding sites (Siersbæk, Nielsen, John, Sung, Baek, Loft, Hager, and Mandrup, EMBO Journal, in press)).

Odom and colleagues have previously shown that the transcription factor binding regions that were retained between multiple species had higher sequence constraint than species specific binding regions [24], however the interdependence between conservation of the region around the bound site and the retention of the consensus site was not addressed. Interestingly, we show here that the high identity quartile of binding sites (i.e. the binding regions with the highest sequence conservation) displayed increased retention of C/EBPα and PPARγ binding independent of conservation of their respective consensus sites. This strongly indicates that additional sequence patterns are important for C/EBPα and PPARγ binding. Such sequence patterns could among others be directing binding of cooperating transcription factors.

## Conclusions

Here we have performed a comprehensive and systematic analysis to investigate what parameters affect the retention of transcription factors binding between the mouse and human genome. We show that retention of PPARγ and C/EBPα binding between mouse and human adipocytes is interdependent, which strongly indicates that these transcription factors cooperate at the level of gaining access to their target sites in the genome. In addition, our data show that overall sequence conservation of the binding region contributes to retention, suggesting that other sequence patterns contribute to retention. Future experimental analyses will be required to dissect the interdependence of PPARγ and C/EBPα and possibly other transcription factors at a molecular level.

## Methods

### Cell culture

The 3T3-L1 fibroblasts were differentiated to adipocytes by stimulation with dexamethasone (DEX), 1-methyl-3-isobutylxanthine (MIX) and insulin as described previously [40]. The cells were harvested for ChIP experiments at day 6 of differentiation. SGBS cells were generously provided by Dr. Martin Wabitsch at the University of Ulm, Germany and differentiated to adipocytes using a procedure modified from previous publications [7,41]. Briefly, SGBS cells were grown to confluence in Dulbecco's Modified Eagle's Medium/Nutrient Mixture F-12 Ham's supplemented with 10% fetal bovine serum, 33 μM biotin, 17 μM pantothenate, 100 μg/ml streptomycin, 62.5 μg/ml penicillin, 1 ng/μl fibroblast growth factors (FGF) 1, and 90 μg/μl heparin. At two days postconfluency, SGBS cells were stimulated to differentiate with serum-free growth medium supplemented with 10 nM insulin, 200 pM triiodothyronine, 1 μM cortisol, 2 μM BRL 49653, 0.115 mg/ml MIX, 0.25 mmol/L DEX, and 0.01 mg/ml human transferrin. After 3 days, the medium was replaced with the differentiation medium without FGF1 and heparin, and after 6 days Rosiglitazone/BRL49653, MIX, and DEX was removed from the medium. The cells were harvested for ChIP experiments at day 10 of differentiation.

### ChIP-seq

ChIP experiments were performed according to standard protocol as described in [42]. Antibodies used were: PPARγ (H-100, sc7196, Santa Cruz), C/EBPα (14AA, sc61). ChIP-seq sample preparation for sequencing was performed according to the manufacturer's instructions (Illumina). Sequence reads from each ChIP-seq library were mapped to the reference genomes mm9 for mouse and hg18 for human using the the BWA alignment software allowing for 2 mismatches [43]. PPARγ and C/EBPα binding sites were identified using MACS [29] with a p-value cutoff of e^-10^, and using sonicated input as a control. C/EBPα and PPARγ ChIP-seqs in 3T3-L1 were performed in monoplicates, and to identify high confidence binding sites we compared our data with previously published C/EBPα and PPARγ ChIP-chip data [16]. ChIP-seq binding regions overlapped by ChIP-chip binding regions were used for downstream analysis. C/EBPα and PPARγ ChIP-seqs in SGBS cells were performed in duplicates from two independent experiments, and binding sites identified in both experiments were used for downstream analysis. Some of the detected sites both in SGBS and 3T3-L1 cells were much wider than expected, possibly due to multiple nearby binding events or noise. To avoid these and focus on reliable sites, we filtered away the 5% widest binding sites.

### Finding regions bound by both PPARγ and C/EBPα

Initial analysis of the overlap between C/EBPα and PPARγ binding sites in mouse showed that in the large majority of cases either most of the site of one factor is overlapped by the site of the other factor or it is not overlapped at all (Additional file 1 Fig. S2). Thus, altering the threshold for how large the overlap should be for sites to be considered overlapping, does not have substantial impact of the downstream analysis. Based on the percentage overlap shown in Additional file 1 Fig. S2 and the length distributions for the binding sites in mouse, a C/EBPα site overlapping a PPARγ site with at least 1% of the total width of the C/EBPα site was defined as "overlapping". The PPARγ binding sites are in general more narrow than C/EBPα, so we used a higher threshold of 10% overlap for PPARγ. In human regions corresponding to mouse sites we relaxed this criterion to 1 nt overlap, since there is an implicit criterion for both sites to be in the same region in the orthology analysis described below. Changing the criteria lead to no substantial difference in terms of overall results (data not shown).

### Finding human genomic regions orthologous to the mouse binding sites

We used the UCSC lift over tool [44,45] together with chained BlastZ alignments [30] to lift over the binding sites from mm9 to hg18 genome assemblies. We used minMatch = 0.1, which is the recommended setting for inter-species comparisons. We then lifted the binding sites back again and tested if they overlapped a minimum of 90% of the original region. Only binding sites that could be uniquely mapped both ways were considered for the rest of the analyses.

### Defining retained, species-specific binding sites and turnover events

For each lifted mouse binding site we checked if >10% of the site was overlapped by a human site, or vice versa. If any of these criteria were true, the mouse site was considered retained. Mouse binding sites that were not retained was considered specific to mouse and lost in human. In the same way, human binding sites that are not overlapped 10% by a lifted mouse binding site or vice versa were considered specific to human.

A lost binding site may have been replaced with a new, species-specific binding site - i.e. binding site turnover. For the binding sites lost in human, we checked if a human-specific binding site is located +/-10 kb (max 10 kb between the midpoints of the two binding sites) from the original lifted binding site. If this was the case, the site was considered subject to a turnover event.

### Calculating ChIP-seq enrichment in human

As a complementary approach to the above, we considered ChIP-seq counts in human regions orthologous to mouse binding sites as a continuous variable rather than counting the overlap of human ChIP-seq peaks defined by the peak finder. This makes sense, as the number of ChIP-seq tags should correspond to the binding strength of a respective factor.

To make this comparable across regions, for a human region defined as orthologous to a mouse binding site, we counted the number of human ChIP tags in a window centered on the region with a window width corresponding to the 75^th ^percentile of all human binding sites, for the respective factor: 520 nt for C/EBPα and 605 nt for PPARγ. We then estimated the number of ChIP-seq tags expected by random by calculating the mean ChIP-seq counts of 2000 randomly sampled genomic regions with the same widths as above. A log_2 _fold change value was calculated by dividing the observed number of ChIP-seq tags in the orthologous region with the random expectation. To avoid small number effects, we added a pseudo-count of 1 to both random regions and observed binding site counts.

### Assigning genes and expression clusters to mouse binding sites

For each binding site we identified all genes (knownGene table from UCSC genome browser database [46]), where the TSS is located +/-100 kb from the center of the binding site. The binding site was labeled with the label of the closest gene that was part of an expression cluster. If none of the nearby genes were in a cluster, the binding site was placed in cluster 5 ("constitutive genes"); whereas the site was considered to belong to cluster 6 ("distal sites") if there were no TSS within 100 kb of the binding site.

### Finding human gene orthologs to the differentially expressed mouse genes

For each differentially expressed mouse gene we used the UCSC lift over tool together with chained BlastZ alignments to lift over the TSS +/-50 bp from mm9 to hg18. We then found the closest gene (knownGene table from UCSC) within 5 kb of the lifted TSS. Using this method we found orthologous genes for 1482 of the 1626 mouse genes. We discarded from further analyses the 113 TSS regions that could not be lifted, and the 49 of the lifted TSSs that were not located in proximity (+/-5 kb) of any UCSC human gene.

### Sequence content analysis

We scanned both strands of each human region defined above with position weight matrices (PWMs) (reviewed in [32]) from the JASPAR database (models MA0065.2 and MA0019.1, respectively, for PPARγ and C/EBPα) using the ASAP tool [47] based on a background model following a uniform distribution. To decide a relevant threshold for accepting a predicted binding site, we repeated the analysis in randomly selected regions with the respective PWM. A threshold of 80% of the scoring range of each model could separate the ChIP'ed regions and random regions best, so we used this for further analysis.

### Calculating human-mouse identity

According to the binding sites in mouse described above, we extracted the alignments between mouse and human by mafFrag from the Kent source utilities http://genomewiki.ucsc.edu. We then applied Alistat, a utility included in the HMMER package, http://hmmer.janelia.org/ to calculate the identity score defined as (#Identity/minimum (length1, length2)), where #identity is the number of exact identities in an alignment and length1, length2 are the unaligned lengths of the two sequences.

### Data availability

Raw sequencing reads, aligned reads, wiggle files, and MACS regions for all ChIP-seq samples described here have been deposited to the NCBI GEO database http://www.ncbi.nlm.nih.gov/geo/ under accession number GSE27450.

## Abbreviations

C/EBP: CAAT/enhancer binding protein; ChIP: chromatin immunoprecipitation; hASC: human adipose stromal cells; MEF: mouse embryo fibroblast; PPAR: peroxisome proliferator activated receptor; PWM: position weight matrices; RNAPII: RNA polymerase II; RXR: retinoid × receptor; SGBS: Simpson Golabi Sehmel syndrom; TSS: Transcription start site

## Authors' contributions

SM, SFS, and AS conceived and designed the study. SFS performed all biological experiments except for deep sequencing, which was performed by RN. MJ and YC performed all computational analysis with help from AS. SFS, MJ, YC and AS made all figures. SFS, SM, AS, MJ and YC wrote the manuscript. All authors read and approved the manuscript.

## Supplementary Material

Additional file 1 **Fig. S1+2.pdf**. Click here for file
