# Supplementary material for: Cross species comparison of C/EBPα and PPARγ profiles in mouse and human adipocytes reveals interdependent retention of binding sites
Source: BMC Genomics. 2011 Mar 16;12:152. doi: 10.1186/1471-2164-12-152 (PMC3068983; doi:10.1186/1471-2164-12-152)
Supplement: Additional file 1 — Fig. S1+2.pdf. [file 1471-2164-12-152-S1.PDF]

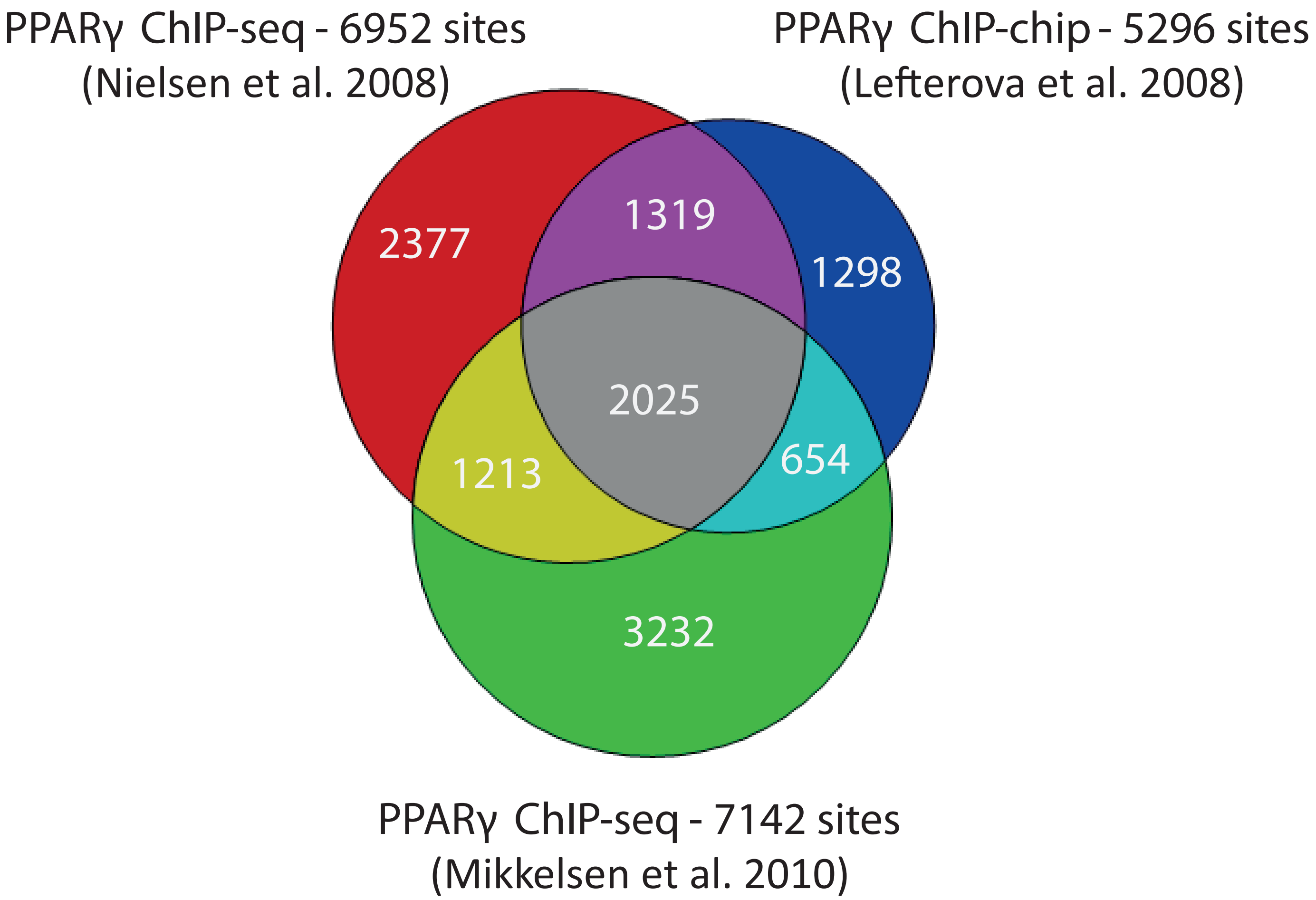

Figure S1. Venn diagram representing the overlap between three genome-wide PPARγ binding studies in 3T3-L1 adipocytes (Nielsen et al. Genes Dev 2008, 22: 2953-2967;Lefterova et al. Genes Dev 2008, 22: 2941-2952; Mikkelsen et al. Cell 2010, 143: 156-169).

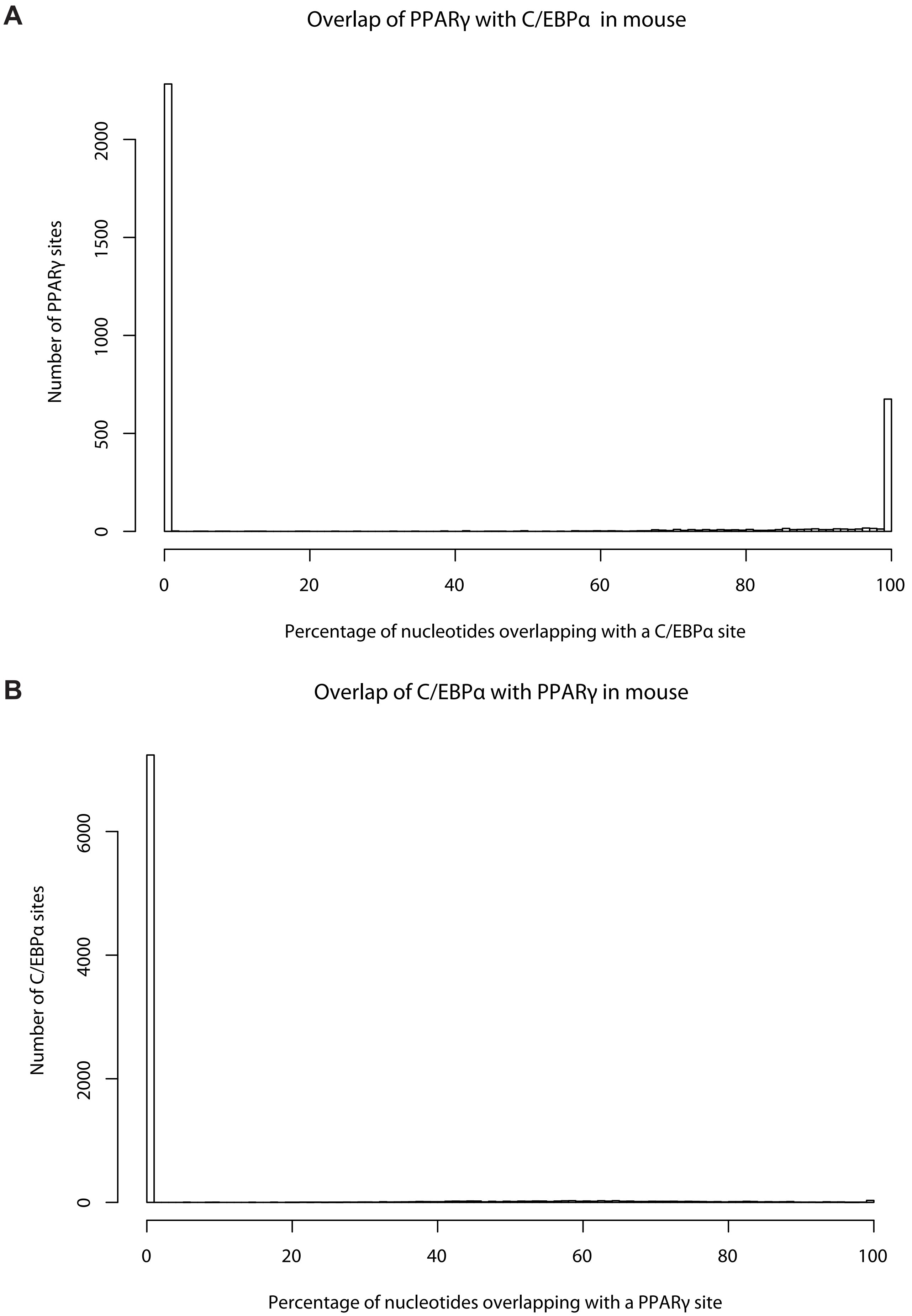

Figure S2. (A) Histogram illustrating the number of mouse PPARγ binding sites (x-axis) that are overlapped by a mouse C/EBPα site with the indicated percentages (y-axis). Most PPARγ binding sites are either overlapped by 100% or not overlapped at all. (B) Histogram illustrating the number of mouse C/EBPα binding sites (x-axis) that are overlapped by a mouse PPARγ site with the indicated percentages (y-axis). Most C/EBPα sites are either overlapped by 50%-100% or not overlapped at all. The overlap by 50-100% is due to the fact that the PPARγ peaks in general are more narrow than the C/EBPα peaks.
